# The Internal Morality of Criminal Law

**DOI:** 10.1093/ojls/gqad005

**Published:** 2023-04-01

**Authors:** James Edwards, Tarek Yusari

**Keywords:** criminal theory, criminal law, philosophy of law, morality

## Abstract

According to a popular picture, criminal law lives up to the demands of its internal morality when its norms have counterparts with the same content in morality—when it conforms to what we call *the mirror principle*. This article argues that the popular picture must be redrawn by relying on a second principle, which we call *the instrumental principle*. Criminal law conforms to the instrumental principle when the existence of its norms helps to prevent or ameliorate moral wrongdoing. Our argument is that the instrumental principle forms part of the internal morality of criminal law, and supplies a justification for criminal laws that depart from the mirror principle. We further suggest that criminal law’s internal morality is asymmetrical: though departures from the mirror principle are sometimes justified by the instrumental principle, departures from the latter are not justified by the former.

## 1. Introduction

Does law have an internal morality? Lon Fuller famously thought so.^1^ Many legal philosophers have agreed.^2^ They have agreed, that is, that there are standards internal to the nature of law, and that law which fails to live up to these standards fails not just as law, but morally too.

Fuller’s claims, of course, are claims about law in general. If true, they identify standards that apply to each area of law. One question to ask of criminal law (and of tort law, contract law, administrative law, employment law, family law, etc) is whether it lives up to standards internal to the genus of which it is a species. A second question is whether there are standards internal to the nature of the species. We can ask, that is, whether part of what it is for a norm to be a criminal law (tort law, etc) is for certain standards to apply to the evaluation of that norm. And we can ask whether norms that fail to live up to those standards fail not just as criminal laws, but morally too. To answer in the affirmative is to claim that, whether or not there is an internal morality of law, there is an internal morality of criminal law.

One popular picture has it that the criminal law lives up to its internal morality when its norms have counterparts with the same content in morality—when those norms conform to what we will call *the mirror principle*.^3^ According to the mirror principle, criminal wrongs should be moral wrongs, moral justifications should be legal justifications, moral excuses should be legal excuses, etc. To subscribe to the popular picture is not to deny that there might be moral justification for divergence: for criminal duties that lack corresponding moral duties, moral justifications that lack corresponding legal justifications, etc. It is only to claim that, if such a justification exists, it is provided by standards that form part of the external morality of criminal law.

In this article, we argue that the popular picture must be redrawn. To do so, we rely on a second principle, which we call the *instrumental principle*. Criminal law conforms to the instrumental principle when the existence of its norms helps to prevent or ameliorate moral wrongdoing. Our argument is that the instrumental principle supplies a justification for divergence—for criminal laws that depart from the mirror principle—that is part of the internal morality of the criminal law.

We make our case by rebutting three objections. According to the first, if the instrumental principle justifies adding a norm to the criminal law, this makes it the case that the addition satisfies the mirror principle. Since the two principles then cannot conflict, the former cannot justify criminal laws that depart from the latter. Call this *the no-conflict thesis*. A second objection assumes what the first denies: that conflict between the mirror and instrumental principles is indeed possible. The objection is that such conflicts ought to be resolved in favour of the mirror principle: where the instrumental principle supports adding a norm to the criminal law but the mirror principle contraindicates, the addition is unjustified, all things considered. Call this *the priority thesis*. A third objection presupposes that the first two fail—that the instrumental principle can justify norms that violate the mirror principle. The objection is that any such justification is nonetheless external. It is a justification provided by a principle that forms part of the external morality of the criminal law. Call this *the externality thesis*.

If any one of these theses is sound, our argument is unsound. If all three are incorrect, so is the popular picture. Section 3 argues that the no-conflict thesis is false. Section 4 makes the case for distinguishing the priority thesis from the externality thesis. It claims that, though the former thesis must be rejected, defenders of the mirror principle need not—and do not—deny this. They can—and do—endorse the externality thesis instead. Section 5 argues that the instrumental principle is part of the internal morality of the criminal law. It follows that the externality thesis is also unsound. If these arguments go through, our conclusion follows: criminal law’s internal morality itself justifies departures from the mirror principle.

Two further preliminary points before we proceed. First, the debate in which we engage in the second half of this article concerns the location of moral standards. All parties claim that, of the set of moral standards to which criminal law is subject, a proper subset is internal to its nature. The remainder of the set are external standards. To make progress with the debate, we need to know how to allocate standards to these two moralities. Call this *the allocative question*. We draw on work in general jurisprudence to identify two answers, then put these answers to work to help defend the conclusions advertised above.

We claim that the instrumental principle forms part of criminal law’s internal morality. Our opponents deny that this is the case. One might reasonably ask what is at stake in all this. Here, Fuller’s discussion of law in general is instructive. As is well known, Fuller claims that law’s internal morality is made up of eight principles.^4^ When he claims that these eight principles are internal, Fuller is not making a claim about their relative moral importance. His claim is not that, when the eight principles conflict with other moral standards, the conflict should always be resolved in favour of the former. Rather, Fuller’s claim concerns the importance of the eight principles for our understanding of the nature of law. His claim is that our understanding of that nature remains impoverished until we understand that law which conforms poorly to the eight fails *qua* law—that it is, to borrow a phrase, in legally poor shape.^5^ Moral standards belong to law’s internal morality as and when succeeding legally requires successful conformity with those standards. Moral standards belong to law’s external morality as and when this is not the case.

If Fuller is right, those interested in understanding law’s nature cannot but be concerned with the answer to the allocative question about law. To neglect that question is to fail to grasp that, among the moral standards to which legal norms are subject, some are standards those norms must meet if they are to be in good shape *qua* legal norms. The significance of the allocative question about criminal law, as we see it, is of essentially the same character. Answering that question matters not because it determines which criminal laws we ought, all things considered, to have. Answering it matters because our understanding of criminal law remains impoverished until we have done so. We might come to know that the norms of the criminal law do nothing to prevent or ameliorate moral wrongdoing. If our opponents are correct, this discovery would do nothing to show that those norms were unsuccessful criminal laws. If our argument in the second half of this article goes through, this is precisely what it would do.

The second preliminary point worth making is as follows. Our aim is not to show that the mirror principle is either unsound or external to the nature of the criminal law. Everything we say is compatible with that principle forming part of criminal law’s internal morality. Our position is that, if this is so: (i) both the instrumental and mirror principles form part of that morality; and (ii) norms which prevent or ameliorate moral wrongdoing can justifiably be added to the criminal law even if those norms lack counterparts with the same content in morality. In short: there are occasions on which our reasons to conform to the instrumental principle provide an internal justification for departures from the mirror principle.

It is worth observing that the truth of (ii) does not entail the truth of the reverse. That is, it does not entail that (iii) norms which have counterparts with the same content in morality can justifiably be added to the criminal law even if the addition will not prevent or ameliorate moral wrongdoing. In short: that an internal justification exists for norms that do not conform to the mirror principle does not entail that an internal justification exists for norms that do not conform to the instrumental principle. Do the arguments on offer for the mirror principle provide a justification of the latter kind? We suggest that they do not. If this is so, and the mirror principle is part of the internal morality of criminal law, that morality is asymmetrical: though departures from the mirror principle are sometimes justified by the instrumental principle, departures from the latter are not justified by the former.

## 2. Two Principles

Let us begin by saying a little more about the two principles that are our subject. According to the mirror principle, the norms of the criminal law should be such that they have counterparts with the same content in morality.^6^ To see what this principle implies, we can usefully distinguish offences from crimes. To commit an *offence* is to commit a criminal wrong: a legal wrong for which one may properly be convicted and punished if one’s conduct does not fall within a legally recognised defence. To commit a *crime* is to commit an offence to which there is no recognised defence: it is to commit a legal wrong for which courts may properly convict and punish wrongdoers. Recognised defences include both justifications and excuses. Roughly speaking, offenders are justified when their reasons for offending are undefeated by those that countervail. They are excused when, though they offend for defeated reasons, doing so discloses no fault in them.

The mirror principle combines a thesis about offences with a thesis about defences. According to the former, legal wrongs that are offences should be moral wrongs.^7^ According to the latter, moral justifications and excuses for offending should be legal justifications and excuses.^8^ Grant that we are morally blameworthy for φing if φing is a moral wrong for which φers have no moral justification or excuse. It follows that a criminal law which conforms to the mirror principle is a criminal law which imposes criminal liability exclusively on the blameworthy.

According to the instrumental principle, crimes should be such that their existence prevents or ameliorates moral wrongdoing. If decriminalising λing would not produce more, or graver, moral wrongs, the instrumental principle implies that λing should be decriminalised. Note that, since the instrumental principle is concerned with the existence of crimes, conformity to that principle depends on both offences and defences. To see this, imagine the legislature adds a new offence to the criminal law. Whether this addition is compatible with the instrumental principle depends on the effects of the legal liability thereby created. Since the extent of that liability depends on the defences available to offenders, its effects are bound up with the scope of those defences.^9^

It is worth noting that the instrumental principle is more capacious than is often supposed. That principle does not oppose the criminalisation of moral wrongs merely because criminalisation fails to prevent or ameliorate those very wrongs. It is compatible with criminalisation that prevents or ameliorates other moral wrongs—other, that is, than the moral wrongs thereby criminalised. This includes moral wrongs that would otherwise be committed in the wake of (actual or supposed) wrongdoing. To take but one example, the instrumental principle is compatible with the criminalisation of burglary if, though criminalising burglary fails to reduce the burglary rate, it succeeds in reducing the rate at which (actual or supposed) victims and their supporters wrongfully retaliate against (actual or supposed) burglars.^10^ We return to this point in section 6.

## 3. Conflict

At first sight, it may seem obvious that the mirror principle and the instrumental principle can conflict. Imagine that criminalising the culpable commission of a moral wrong will not reduce the incidence or gravity of moral wrongdoing. Criminalisation is then compatible with the mirror principle but not with the instrumental principle. Alternatively, imagine that neither the incidence nor the gravity of moral wrongdoing will be reduced unless we criminalise what is not morally wrong. Criminalisation is then compatible with the instrumental principle but not with the mirror principle. What we earlier called the no-conflict thesis is straightforwardly false.

Some argue, however, that this is too quick. Imagine a legislature that is considering whether to make it a criminal offence to φ. Absent legislation, two things are true: (i) it is not morally wrong to φ; and (ii) there will be more moral wrongs than there would be if the legislation were enacted. Now the moral reason to legislate in this way is either defeated or undefeated. If the former, our legislature should not act. If the latter—so the argument goes—the legal duties imposed by the legislation are morally justified. And if a legal duty is morally justified, that legal duty is morally binding. Moral justification in. Moral duty out. Here is John Gardner:

All crimes are legal wrongs and all legal wrongs are purported moral wrongs, i.e. they are moral wrongs according to the law. All that it takes to turn them into true moral wrongs is that the law in question is morally acceptable.^11^

More generally, ‘Some legal norms impose obligations, and when they do so and they are morally justified in doing so, they impose moral as well as legal obligations’.^12^ Call this *the justification in, duty out principle*. JIDO, for short.^13^ Grant, for a moment, that JIDO is sound. Now imagine that our legislators are justified in legislating by the preventive benefits of the legislation. The instrumental principle is therefore satisfied. So, JIDO tells us, is the mirror principle: because the offence created by the legislation is morally justified, it is morally wrong to commit it. It follows that, at least when it comes to the creation of criminal offences, the no-conflict thesis is sound: if adding a criminal offence satisfies the instrumental principle—given the scope of the available defences—the addition necessarily also satisfies the mirror principle.

Is JIDO sound? We will argue that it is not.^14^ To see why, we can start by considering two sets of facts: (i) the facts which make it the case that legal duties are morally justified; and (ii) the facts which make it the case that legal duties are morally binding on those to whom the duties apply. JIDO is sound only if the relationship between (i) and (ii) is one of identity or entailment. The relationship is one of identity if the facts that confer moral justification upon legal duties are necessarily also facts that suffice to make those duties morally binding. The relationship is one of entailment if the existence of facts that confer moral justification upon legal duties necessitates the existence of other facts that suffice to make them morally binding.

That neither relationship holds is a function of two further facts: (iii) the fact that we are fallible users of norms: that we are disposed to error in applying norms to which we are subject; and (iv) the fact that we are unequally fallible: that we are disposed to make different errors in the application of different norms. We can see why these facts matter by considering an example. Imagine that P and Q are both engaged in activities that put others at risk of harm. They are, let us say, manufacturers of drugs used to treat serious disease. Both could make use of a manufacturing process that would allow them to produce drugs more cheaply. If that process is inexpertly used, however, the drugs will cause serious harm to patients. P has the expertise to use the process safely. Q lacks this expertise, but erroneously believes things to be otherwise. Given the seriousness of the harm done, Q commits a moral wrong—a wrong of endangerment—by making use of the cheaper process: Q’s activities expose patients to a risk of harm for reasons that fail to justify the exposure.

Imagine next that there are many manufacturers like P and many like Q. We cannot reliably distinguish in advance between those who have and those who lack the expertise to use the cheaper process safely. One option is to make the moral wrong Q commits a criminal offence. Call this *the dangerous use option*. Given the fallibility of manufacturers like Q, the protective effect of this option would be limited. *Ex hypothesi*, these manufacturers erroneously believe their activities to be safe. They are therefore unlikely to refrain from using the cheaper process merely because it is a criminal offence to use it dangerously. A second option is to make using that process an offence in all cases. Call this *the all use option*. Though manufacturers like Q are unreliable judges of whether their actions are dangerous, we can assume that they are reliable judges of which processes they employ. We can also assume that they tend to be motivated to avoid criminal liability. Under these assumptions, the all use option would offer greater protection for patients than the dangerous use option. It might be said that the all use option is compatible with retaining some of the benefits of the cheaper process by making a defence of safe use available for manufacturers like P. Given the erroneous beliefs held by manufacturers like Q, however, this defence would risk undercutting the protective effects of the offence. The same tendency to error that counts in favour of preferring the all use option to the dangerous use option also counts against combining the former with a defence of safe use.

Grant that the all use option—absent a safe use defence—would result in fewer wrongs of endangerment than the available alternatives. It does not follow, of course, that a legislature would be justified in so legislating. It is nonetheless hard to believe that it never could be—that preventing serious harm to a sufficiently large number of patients could not justify using the criminal law to obligate P and Q to use a more expensive manufacturing process. JIDO tells us that where legislators impose this obligation, and are justified in doing so, both P and Q commit a moral wrong by using the cheaper process.

The problem with JIDO is not, of course, the conclusion it reaches about Q. The problem is the conclusion JIDO reaches about P. Recall that the fact which justifies imposing a legal obligation on P is the fact that Q will act wrongly absent that obligation: if an exception is made for P, Q will erroneously take that exception to apply to her own activities, and those activities will unjustifiably endanger others. In short, Q’s greater fallibility justifies P’s legal obligation. But the fact that someone else will act wrongly if I am not legally obligated to refrain from φing is not a reason for me to be morally obligated to refrain. Nor does it entail the existence of such a reason. This is not to deny that, if my violating a legal obligation would encourage others to violate their moral obligations, this may make it the case that my legal obligation is morally binding on me. Nor is it to deny that, if conforming to a legal obligation would improve my conformity with moral obligations, I may be morally obligated to conform to my legal obligation on these grounds.

Neither of these facts figures in the justification of the duties imposed on P in our example. The reason to obligate P to refrain from using the cheaper process is not the fact that Q will use that process if P uses it. It is the fact that Q will use that process if P is permitted to use it. Nor is it true that P’s conformity with P’s legal obligation would improve P’s conformity with P’s moral obligations. Since P can use the cheaper process safely, and since P using it reduces the cost of treating serious disease, P has a weighty moral reason to *violate* any legal obligation not to use that process. Be that as it may, the key point is this: the fact that justifies P’s legal obligation is not that P violating that obligation would set a bad example. Nor is it that P conforming to the law would serve P. The justification for P’s obligation is simply this: that Q would take P having a legal permission to act morally permissibly as an inducement to moral wrongdoing. No doubt this is a reason for legislators to deny P the legal permission. It is no reason to conclude that, if the permission is denied, P is also denied the moral permission from which P otherwise benefits.^15^

## 4. Stringency

We conclude that JIDO cannot stand. The facts that justify making it a criminal offence to φ do not always justify a moral duty not to φ. Nor do they entail the existence of such a duty. There are cases in which criminal offences justified by the instrumental principle are not criminal offences it is morally wrong to commit.^16^ It follows that the no-conflict thesis must be rejected. It also follows that the same is true of the priority thesis. According to that thesis, where the instrumental principle supports adding a norm to the criminal law but the norm does not conform to the mirror principle, the addition is necessarily unjustified, all things considered. This thesis is false if—as we are have argued—law makers can be justified in criminalising morally permissible conduct of P on the ground that doing so is necessary to prevent conduct of Q that is morally wrongful.

Rejecting the priority thesis may seem a bold and contentious move. We doubt, however, that it really is. It is true that defenders of the mirror principle are sometimes associated with that thesis. But we know of no philosopher of criminal law who insists upon it. What some do insist upon is the truth of the externality thesis. According to the latter thesis, while moral justification for departing from the mirror principle can be found, that justification forms part of the external morality of the criminal law.

We can usefully distinguish, at this point, between two ways of thinking about the stringency of moral principles. Let us say that a standard is absolute if other standards cannot justify failing to live up to it. A standard is defeasible if other standards sometimes justify the failure. As these remarks imply, conclusions about stringency are conclusions relative to a set of standards. What we conclude about the stringency of *s* for X depends on which standards we include in the set. One option is to include both the internal and the external moralities of X. This gives us an *all-things-considered* assessment of the stringency of *s* for X. Another option is to take account only of the internal morality of X. This gives us an *internal* assessment of the stringency of *s* for X. Now imagine *s* is a standard which forms part of X’s internal morality, and that X’s failure to live up to *s* can only be justified by external moral standards. It then follows that, in its application to X, *s* is defeasible, all things considered. It is also internally absolute.

Which type of assessment is offered by defenders of the mirror principle? The textual evidence is admittedly mixed. Some passages do suggest that an all-things-considered assessment is on offer.^17^ They lead critics of the mirror principle to object that, since violation of the principle can be morally justified, it is a mistake to regard that principle as absolute.^18^ We have just seen that this is too quick if the assessment being offered is internal. The moral standards that justify violating the mirror principle might yet form part of the external morality of criminal law. The mirror principle might yet be absolute internally.

Defenders of the mirror principle are sometimes explicit about their objectives. Where they are, they tend to claim that it is an internal assessment that is on offer. Michael Moore, for instance, writes that his aim is to provide an ‘internal justification’ of the criminal law. Such a justification brackets ‘external considerations’ and concerns itself only with ‘the good(s) an institution can achieve that are the reason(s) we have for creating it’.^19^ Antony Duff writes that he is interested in ‘what criminal law ought to be, if it is to be true to the aims and values that are internal to it as a distinctive legal institution’.^20^ And Doug Husak argues for several ‘internal constraints’ on criminalisation—with explicit reference to Lon Fuller—to be defended ‘by attending to the nature of the criminal law’.^21^ All accept that there can be cases in which violating the mirror principle is all-things-considered justified.^22^ What they deny, it seems to us, is that the considerations which justify the violation form part of the internal morality of the criminal law. This is to endorse the externality thesis.

If we are right so far, discussion of the morality of the criminal law should distinguish more clearly between two debates. The first takes account of criminal law’s external and internal moralities. The second suppresses the former to clarify the content of the latter. Which debate one chooses to engage in depends on one’s philosophical interests. Some philosophers are primarily interested in understanding what we are morally permitted to do with the legal instruments at our disposal, once all morally relevant considerations that bear on their use are factored in. Others are primarily interested in understanding the nature of those instruments, and in what follows for how they are permissibly used when all else is equal. It is because the internal morality of criminal law is made up of standards internal to the nature of criminal law that those who pursue the latter interest focus on that morality. It is the focus of our discussion in the remainder of this article.

## 5. Location

We turn now to the externality thesis. To determine whether that thesis is true, we need to answer a prior question. The question is how we draw the line between standards internal to the nature of the criminal law and those external to that nature. This is what we earlier called *the allocative question*. We begin by outlining two answers drawn from general jurisprudence. We note that, though their terminology is not ours, philosophers of criminal law use both answers to argue that the mirror principle is part of criminal law’s internal morality. We then use one answer to reject the externality thesis. If we are right, then, whatever the status of the mirror principle, the instrumental principle is internal to the nature of the criminal law.

### A. Ends and Means

This article offers an argument. We hope it is free of *non sequiturs*. Imagine, however, that we are mistaken. Our argument is then defective in argumentative terms. It falls short of a standard internal to the nature of arguments. It does not follow, of course, that this defect is a moral defect. It is one thing for *s* to be internal to the nature of X. It is another for *s* to be a moral standard. Our principal question is not whether the mirror and instrumental principles are moral principles. Rather, it is why we should think that criminal laws which fail to conform to them are akin to arguments that harbour *non sequiturs*—why we should think of such criminal laws, whether or not they are morally defective, as defective *qua* criminal laws.

Two answers are on offer to the allocative question when it comes to law in general. One is telic. The other is modal. The former answer appeals to law’s *telos*—to the end(s) that give us reason(s) to bring law into existence and maintain it as such.^23^ Standards law must meet to serve those ends are internal to its nature. The latter answer appeals to law’s *modality*—to the means we use to (try to) achieve our ends when we use law to (try to) achieve them. Law is defective *qua* law when it falls short of standards brought to bear by those means.

As is well known, Lon Fuller and Joseph Raz offer (versions of) the modal answer. Both argue that standards of usability are internal to the nature of law. Such standards include standards of clarity, stability, publicity, consistency, etc. They are standards laws must meet if they are to be used as guides by those subject to them.^24^ Why think these standards internal? Because—to borrow Fuller’s well-known phrase—law is a means of achieving ends by subjecting human conduct to the governance of rules.^25^ Since this is law’s modality, and since rules are defective *qua* rules if they cannot be used as guides, law is defective *qua* law if it falls short of standards of usability.

Ronald Dworkin and John Finnis develop versions of the telic answer. For Dworkin, law’s *telos* is to morally justify the government’s use of coercion, by subjecting that use to standards found in past political practice.^26^ For Finnis, law’s *telos* is to contribute to the common good, by helping co-ordinate the behaviour of legal subjects.^27^ On the former view, what it takes for law to serve its *telos* depends on the morality of coercion. According to the latter, it depends on the moral character of the common good. On either view, law that falls short of these moral standards—that fails to justify coercion or to serve the common good—is properly regarded as degenerate or deviant law.^28^ It is properly so regarded because it fails to serve the ends that justify creating and maintaining law as such.

The modal and telic answers are not incompatible. One can combine the two. Strictly speaking, both Dworkin and Finnis do just that. They attribute to law both a justifying end and a particular means of contributing to its realisation. There are various ways of contributing to the common good. Law’s own way is to contribute by co-ordinating behaviour. There are various ways of contributing to the moral justification of coercion. Law’s own way is to contribute by having past political practice constrain its use. On these conjunctive views, law’s internal morality consists of two sets of standards: those law must meet to contribute to ends in its own way; and those it must meet for its contributions to serve law’s own end(s).

One debate about law’s internal morality concerns the content of the standards that make up the first of these two sets. A second debate concerns whether the second set of standards exists at all. We will deny that it does if we deny that law as such has a *telos*. This, as we understand it, was HLA Hart’s position. For Hart, there is no ‘specific purpose which law as such serves beyond providing guides to human conduct and standards of criticism of such conduct’.^29^ Some take Hart to be claiming here that law’s *telos* is to guide. But guiding people is not an end in itself. It is a means for the achievement of ends. There is nothing to be said for guiding people away from some course of action if no further valuable objective is achieved by doing so.^30^ Hart is not denying, of course, that various areas of law achieve various valuable ends by means of legal guidance. Nor is he denying that legal systems must achieve at least some of these ends if their creation and maintenance is to be morally justified. He is only denying that law has *unifying* ends—ends that justify creating and maintaining law as such. For Hart, law is unified not by a *telos*, but by a modality. Law as such *has* no telos.

To claim that the telic answer is inapplicable to law in general, of course, is not to reject that answer outright. It is not to deny that, if a practice has a *telos*, and if that *telos* will not be realised unless certain moral standards are met, those standards form part of the internal morality of the practice. Nor, given what we have said so far, could such a denial be sustained. Moral standards form part of X’s internal morality, we have claimed, if conformity to those standards is necessary for that practice to succeed in its own terms. A practice with a *telos* succeeds in its own terms only if that *telos* is realised. It follows that moral standards the practice must meet to realise its *telos* are standards that form part of its internal morality. Our argument below is that, in the case of the criminal law, those standards include the instrumental principle.

### B. Locating the Mirror Principle

More on this momentarily. Notice first that both the modal and telic answers are used by philosophers of criminal law to contend that the mirror principle is internal to its nature. As to the former, consider what we might call *the argument from censure*. The argument’s first premise is that criminal law is a means of achieving ends that is censorious. Put simply, convicting D of having committed a crime involves censuring D for its commission. The second premise is that censuring D for a crime is an act of moral communication: it communicates both that D’s offending act was morally wrong and that D is morally blameworthy for its commission. The third premise is that criminal law’s modality brings to bear a truth constraint. Criminal law should not communicate that offending acts are moral wrongs unless this is indeed what they are. It should not communicate that offenders are morally blameworthy for their crimes unless committing those crimes indeed makes them blameworthy.^31^ This is so only if moral justifications and excuses are available defences to D’s offence. It follows, assuming all this is correct, that the truth constraint entails the mirror principle. It also follows, if the modal answer is correct, that the mirror principle is internal to the nature of the criminal law.

So much for means. Now consider ends. Michael Moore claims that criminal law’s *telos* is to do retributive justice.^32^ Antony Duff claims that its *telos* is to call public wrongdoers to public account for those wrongs.^33^ Deriving the mirror principle from either end requires a series of further steps. In Moore’s case, the first is that retributive justice is done by punishing the deserving for that which makes them deserving of punishment. The second is that what makes us deserving of punishment is the blameworthy commission of moral wrongs. The third is that criminal law punishes offenders for the blameworthy commission of moral wrongs—thereby doing retributive justice—only if criminal offences are moral wrongs, and only if moral justifications and excuses are defences to those offences.^34^

In Duff’s case, the route is different. For our purposes, however, the conclusion is much the same. The first step is that, though not all moral wrongs are public, all public wrongs are moral. The second is that calling another to account for a public (or any) wrong involves engaging (or attempting to engage) the wrongdoer in dialogue. It involves putting the accusation of wrongdoing to the (alleged) wrongdoer and offering them the opportunity to explain themselves by justifying or excusing its commission. The third step is that the accusation put to defendants in criminal proceedings is that they committed an offence. The opportunity they are given to explain themselves is that of pleading available defences.^35^ It follows that criminal proceedings call public wrongdoers to account for those wrongs when two conditions are met. First: criminal offences are themselves public wrongs. Second: moral justifications and excuses for those wrongs are defences to those offences. If all this is right, criminal law’s *telos* calls for conformity to the mirror principle. If the telic answer is right, that principle is internal to the nature of the criminal law.

### C. Locating the Instrumental Principle

Duff and Moore claim that criminal law’s telos is both monistic and responsive. It is *monistic* in that it consists of a single justifying end. It is *responsive* in that the end in question is served by responding to offending acts—by imposing deserved punishment on those guilty of crimes, or by calling public wrongdoers to public account for criminal offences. Let us call the conjunction of these claims *the responsive view*.

This subsection argues that the responsive view should be rejected. It does so by arguing that criminal law’s *telos* is at least partly preventive: that our reasons to create and maintain systems of criminal law include reasons to prevent or ameliorate moral wrongdoing. If our argument is sound, the telic answer implies that we must reject the externality thesis. It implies that the instrumental principle is internal to the nature of the criminal law.

We can usefully begin by imagining a society in which the result of establishing a system of criminal law is that those subject to its norms conform to them without exception. All those who would otherwise have committed moral wrongs of legitimate interest to the criminal law—murder, rape, dangerous driving, insider trading, burglary, theft, and so on—refrain from doing so as a consequence of their criminalisation.

It is worth pointing out that our imagined society is not Joseph Raz’s society of angels:^36^ it is not a society all members of which conform to legal rules merely in virtue of the fact that they are rules. Rather, it is a society closer to that described by Hart: a society in which members tend to conform to the law for reasons prudential and moral, but in which

neither understanding of long-term interest, nor the strength or goodness of will, upon which the efficacy of these different motives towards obedience depends, are shared by all [members] alike. All are tempted at times to prefer their own immediate interest [such that] in the absence of a special organisation for their detection and punishment, many would succumb to the temptation [to disobey].^37^

In this society, the prospect of conviction and punishment serves ‘not as the normal motive for obedience, but as a *guarantee* that those who would voluntarily obey shall not be sacrificed to those who would not’.^38^ Our imagined society differs from Hart’s only in that the guarantee turns out to be exceptionless: criminal law’s existence results not just in fewer murders, rapes, thefts, burglaries, etc, but in the eradication of these and other criminal wrongs.

Recall next that, according to the responsive view, criminal law’s *telos* is served by criminal proceedings conducted in response to offending acts. In the society we have imagined, there *are* no offending acts for which criminal proceedings might be conducted. So there is no occasion for our society’s criminal law to do retributive justice, or to call public wrongdoers to public account. We know that the *telos* of a practice supplies the reasons to create and perpetuate its existence. It appears to follow, on the responsive view, that our imagined society has no reason to perpetuate its system of criminal law. Since it is impermissible to perpetuate a costly system absent reason to perpetuate its existence, it also seems to follow that abolition of the system is morally required. This, clearly, cannot be right. All else being equal, any society possessed of such a criminal law would have decisive reason to retain it, a reason supplied by the preventive effects of the system’s existence.^39^ Far from acting wrongly by perpetuating criminal law’s existence, our society would commit a grave wrong by abolishing it.

It may be objected that the guarantee we are imagining could not hold for a prolonged period. Soon enough, there would be murderers, thieves and other wrongdoers to try and punish. So be it. The responsive view implies that criminal law cannot succeed *qua* criminal law until this is the case. Absent criminality, there is no occasion for it to conform to the reasons that justify its existence.^40^ The point of our example is to suggest that this cannot be right. The criminal law of our imagined society, we are claiming, is not a criminal law as yet incapable of succeeding. It is a criminal law that could not be more successful.

It may be said in reply that this is too quick. While the *telos* of a practice supplies reasons to perpetuate its existence, not all reasons to perpetuate a practice’s existence form part of its *telos*. Consider, by way of example, the sport of basketball. We might discover that, in cities where basketball is played, there are fewer instances of violent crime between citizens. We might also discover that the former is among the causes of the latter. All else being equal, the fact that basketball made this contribution, in cities in which it was played, would be a decisive reason to perpetuate its existence as a sport. We might still doubt that preventing violent crime is part of the *telos* of the sport of basketball. We might doubt this because basketball’s preventive effects, while of great moral importance, do nothing to account for the content of the rules that constitute the sport. While those effects are reasons to encourage participation in a sport constituted by the rules of basketball, they do not figure among the reasons why the sport is constituted by those rules. The *telos* of basketball—and, we might add, any other rule-constituted practice—is made up of such explanatory reasons. Preventing crime is no part of the *telos* of basketball, the thinking goes, because it fails this explanatory test.

Should the same be said of the criminal law? Moore answers in the affirmative. He claims that prevention fails to account for the doctrines constitutive of Anglo-American criminal law, and that retributive ends alone explain the existence and content of those doctrines.^41^ Grant both that this is true, and that the explanatory test is a sound one. The preventive effects of criminal law are then like (what we imagined to be) the preventive effects of basketball: neither form part of the *telos* of the practice that produces them.

To respond to this line of thought, let us begin with two points. First, our argument here is not that one monistic view should be replaced with another. Our thesis, recall, is that preventive ends form part of the *telos* of the criminal law. On a pluralistic view, this is true of responsive ends too. Imagine, then, that we need to invoke both ends to pass the explanatory test. That test then supports, rather than undermines, our thesis.

Second, Anglo-American criminal law consists of doctrines of two types. Some are substantive. These doctrines determine conditions of criminal liability of general application within the system. Other doctrines are procedural. They determine what can lawfully be done, both by officials and private persons, to address conduct that violates the conditions of criminal liability.

It is procedural doctrines that provide the most obvious reason to doubt Moore’s doctrinal claim. Consider, for example, section 3 of the Prevention of Crime Act 1967: ‘A person may use such force as is reasonable in the circumstances in the prevention of crime, or in effecting or assisting in the lawful arrest of offenders or suspected offenders or of persons unlawfully at large.’

Forcibly arresting suspected offenders, or persons unlawfully at large, can serve responsive ends—it can facilitate wrongdoers being called to public account, or given their just deserts. The legal permission to use such force can therefore be explained by the responsive view. The legal permission to use force to prevent crime cannot be. There are many other similar examples.^42^

It might be said in reply that, since our focus is on principles that govern the substantive law, it is substantive doctrines that are of sole relevance here. Be that as it may, the same point can be made about such doctrines. It is commonly claimed (including by Moore)^43^ that P φing is morally wrong, or that P is blameworthy for φing, only if (i) P φs with *mens rea* and (ii) P lacks a moral justification or excuse for having φed. Grant that this is so. If Moore’s doctrinal claims were correct, we would expect English law to contain doctrines that made (i) and (ii) conditions of criminal liability. If such conditions are in fact absent, and if the justification for their absence is preventive, we have reason to reject Moore’s claims. We have reason to accept that, far from failing the explanatory test, views that make prevention part of the *telos* of the criminal law are the only ones that pass it.

Let us start, then, with (i). English law contains a well-established presumption of *mens rea*: it is presumed by the courts that every criminal offence is partly constituted by an element of *mens rea*. This presumption can be, and often is, overridden. In *Gammon*, the Privy Council laid down five general principles to be used in determining whether this is so. According to what we can call the efficacy principle,^44^

the presumption of mens rea stands unless it can also be shown that the creation of strict liability will be effective to promote the objects of the statute by encouraging greater vigilance to prevent the commission of the prohibited act.

Courts should also consider the severity of the available punishment, whether offending acts imperil public safety and whether those acts are part of specialist activities in which most of us do not engage. Satisfaction of the efficacy principle is far from sufficient to rebut the presumption. But *Gammon* shows us that, when courts decide whether *mens rea* elements are among the conditions of criminal liability, they take a set of principles into account. The efficacy principle shows us that the set cannot be accounted for without reference to preventive considerations. Far from supporting the truth of Moore’s doctrinal claim, the presumption of *mens rea* therefore helps to call it into question.

Next consider (ii). Whether we are blameworthy for criminal conduct depends on whether we are *morally* justified or excused. It is often the case that, where this is so, D’s conduct is also justified or excused *legally*. But the legal and moral categories are far from co-extensive. We can see this by considering two cases of justification.

The first case is that of self-defence. English law, like many other jurisdictions, makes the availability of this defence subject to an imminence requirement:^45^ D may use force to prevent an attack threatened by V only if what V threatens will take place imminently. We are not always blameworthy for using force to defend against non-imminent threats. Imagine V kidnaps D and announces that V will kill D in a week. D has the opportunity to kill V each morning when V brings D her daily gruel.^46^ Morally speaking, D need not wait until V is standing over D with a knife. D is morally justified in killing V if this is the only way D can escape her fate. What accounts for the imminence requirement is that it helps prevent offending that turns out *not* to be justified. As Baron writes:

The imminence requirement provides a safeguard of sorts: in insisting that self-defensive killing be limited to thwarting an imminent attack, we reduce the risk that a life will be taken needlessly. If the attack is not imminent, there is always the hope that even without the (presumedly) intended victim taking steps to thwart it, it will not happen. Maybe the aggressor will change his or her mind; maybe he or she was not really intending to inflict harm but just to scare or intimidate; or perhaps the whole thing was intended as a harmless prank.^47^

What accounts for the imminence requirement, then, is that it helps prevent offending acts that—because needless—turn out to be morally unjustified. We have seen that the requirement *also* criminalises some acts that are morally justified. It follows that the imminence requirement opens a first gap between moral and legal justification, a gap accounted for—at least in part—by the preventive considerations Baron describes.

Our second case is that of lesser evils necessity. Imagine a family living on the street in the heart of winter. Having pursued all lawful means of obtaining accommodation, they take shelter in unoccupied premises, then burn some furniture to make a fire. It is hard to believe that this is not morally justified, or at least morally excused. English law nonetheless offers no defence to our family. According to Lord Denning MR,

if hunger were once allowed to be an excuse for stealing, it would open a way through which all kinds of disorder and lawlessness would pass. So here. If homelessness were once admitted as a defence to trespass, no one’s house could be safe. Necessity would open a door which no man could shut. It would not only be those in extreme need who would enter. There would be others who would imagine that they were in need, or would invent a need, so as to gain entry. Each man would say his need was greater than the next man’s. The plea would be an excuse for all sorts of wrongdoing. So the courts must, for the sake of law and order, take a firm stand.^48^

If a general defence of necessity were available, Lord Denning here claims, some would offend in error (those whose need was imagined) and others would exploit the availability of the defence (those whose need was invented). The absence of the general defence from English criminal law is accounted for, at least in part, by the value of preventing these offending acts.

Much the same point is made in *Dudley and Stephens*. The defendant sailors—shipwrecked, starving and with no rescue in sight—killed and ate their ship’s cabin boy to prolong their own lives. Tried for murder, they argued necessity. As is well known, the defence was refused. One of the court’s reasons for refusal appeals directly to the moral quality of the sailors’ acts. Their defence should fail, says Lord Coleridge CJ, because what they did was morally unjustified:

Though law and morality are not the same, and many things may be immoral which are not necessarily illegal, yet the absolute divorce of law from morality would be of fatal consequence; and such divorce would follow if the temptation to murder in this case were to be held by law an absolute defence of it.^49^

Lord Coleridge does not here say that killing an innocent can *never* be morally justified: the words ‘in this case’ leave open that it might be. Anyone who accepts that the proverbial trolley can permissibly be turned accepts that such cases exist. Should a general defence of necessity be created to cater for them? Not for Lord Coleridge. We should instead heed the words of Milton:

So spake the Fiend, and with necessity

The tyrant’s plea excused his devilish deeds.^50^

In quoting these words, Lord Coleridge does not mean to accuse the sailors of deeds that are devilish. He means to say that any court considering a general defence of necessity must reckon with its potential to unleash devilishness in cases yet to come. For Lord Coleridge, ‘it is quite plain that such a principle once admitted might be made the legal cloak for unbridled passion and atrocious crime’. This ‘awful danger’ is, for him at least, sufficient reason why no general defence should be available—why legal and moral justification should again come apart.^51^

There is, of course, much more to say about how best to account for Anglo-American criminal law doctrine. Enough has been said, however, to show that the explanatory test is no friend of the responsive view. Far from casting doubt on the claim that prevention is part of the *telos* of the criminal law, the explanatory test supports the conclusion that it must be. Since we can account for English criminal law doctrine only by reference to preventive considerations, that test implies that those considerations cannot be excluded from its *telos*. If they cannot, the telic answer does not support the responsive view. On the contrary, it supports the conclusion that the instrumental principle is part of the internal morality of the criminal law.

## 6. Asymmetry

Section 5 completes our argument against the three theses with which we began. Against the no-conflict thesis, we argued that the instrumental principle can conflict with the mirror principle. Against the priority thesis, we claimed that the former can justify departing from the latter. We also claimed that this is widely accepted. Against the externality thesis, we argued that the instrumental principle forms part of the internal morality of the criminal law.

It is no part of our argument that the same cannot be said of the mirror principle. Grant that it can. Our argument then implies that (i) criminal law’s internal morality contains standards that come into conflict and that (ii) one of these standards—the instrumental principle—sometimes justifies departing from another—the mirror principle. One question we have not addressed is whether the same is true in reverse—whether the reasons for the criminal law to conform to the mirror principle are reasons that sometimes justify departing from the instrumental principle. If they do not, and the mirror principle is indeed part of the internal morality of criminal law, that morality is asymmetrical: though departures from the mirror principle are sometimes justified by the instrumental principle, the reverse is not true. Call this *the asymmetry thesis*. Call its denial *the symmetry thesis*.

Which thesis we should accept depends on the arguments on offer for the mirror principle. Arguments for that principle support the symmetry thesis only if they pass three tests. First, they must identify a reason to add norms to the criminal law. Second, that reason must support the addition of norms that conform to the mirror principle. Third, the reason for the addition must not be that this will prevent or ameliorate moral wrongs.

We already know that one argument for the mirror principle is modal. The argument is that, given criminal law’s censorious modality, norms of the criminal law should conform to a truth constraint. Conforming to that constraint, so the argument goes, necessitates conforming to the mirror principle.^52^ This argument from censure fails our first test. It implies that *if* we have reason to add norms to the criminal law, those norms should not speak falsely about the convicted and punished. It does not itself identify any reason for the addition. It therefore fails to identify a justification for adding norms to the criminal law that neither prevent nor ameliorate moral wrongdoing.^53^

A more promising argument for the symmetry thesis is telic. Let us grant that Moore and Duff are right to the following extent: (i) our reasons to create and maintain systems of criminal law include reasons to respond to wrongdoing; (ii) these reasons are reasons to do retributive justice, or to call public wrongdoers to public account for their wrongs; and (iii) criminal proceedings contribute to conformity with these reasons if and only if the norms of the criminal law conform to the mirror principle.

This argument, if sound, passes our first two tests. At least as Moore and Duff understand the argument, however, it fails the third. Take the retributive version first. Retributive justice is a type of justice, Moore claims, precisely because we have moral duties to pursue it.^54^ Our reason to add norms to the criminal law that conform to the mirror principle is that these moral duties will be breached more often in the absence of said norms. Fewer of the deserving will get the punishment they deserve and more of the undeserving will suffer punishment of which they are undeserving. Making it the case that a moral duty is breached less often just is preventing a moral wrong. It follows without further ado that Moore’s retributive argument cannot justify departures from the instrumental principle. It cannot do so because the reason it gives us to conform to the mirror principle is that conformity will prevent moral wrongdoing.

Much the same can be said about Duff’s telic argument. Duff claims that political communities have moral duties to call public wrongdoers to account in criminal courts and that public wrongdoers have moral duties to account for their wrongs when called.^55^ Our reason to add norms to the criminal law—norms that, we are assuming, must conform to the mirror principle—is that doing so prevents violations of these moral duties. Note that this is not to ignore the distinction between discharging a duty and preventing a wrong.^56^ P φing discharges a duty if φing just is what P has a duty to do. P φing prevents a wrong if Q has a duty to μ that Q would otherwise breach and one of the results of P φing is Q μing. For our purposes, to φ is to add norms to the criminal law; to μ is to bring criminal proceedings that call public wrongdoers to account for their wrongs. We claim that if (i) our reason to μ is that μing discharges a moral duty and (ii) our reason to φ is that there are instances of μing that will not occur unless we φ, then (iii) our reason to φ is a reason to prevent a moral wrong. Criminalisation justified by such a reason conforms to the instrumental principle. An argument that relies on it cannot justify departing from that principle.^57^

None of this, of course, amounts to a knockdown argument against the symmetry thesis. What it does show is that the reasons to conform to the mirror principle identified by some leading arguments in its favour do not justify departing from the instrumental principle. We have argued that things are different in reverse. Our reasons to conform to the instrumental principle do sometimes justify departing from the mirror principle. The leading arguments may not, of course, be the best arguments. The mirror principle may not be internal to the nature of the criminal law. But if they are, and it is, we should accept the asymmetry thesis: we should accept that, though departures from the mirror principle are sometimes justified by the instrumental principle, the reverse is not true.

## 7. Conclusion

It is one thing to prevent a wrong. It is another to respond to its commission. Responding to one wrong may nonetheless prevent another. And the two may have a common foundation. The reasons why we have duties not to commit murder or theft may be among the reasons why we have duties to try or punish murderers and thieves. Where this is so, failing to try or punish wrongdoers is a secondary moral wrong. It is the wrong of breaching a duty that exists in part because of the breach of another duty.

It should go without saying that preventing secondary moral wrongs is morally second best. All else being equal, it is morally better to prevent murder and theft than to prevent the wrong of murderers and thieves murdering and thieving with impunity. On what we earlier called the responsive view, things are different for the criminal law. Success for the criminal law, on that view, consists in discharging secondary moral duties—in doing retributive justice or calling public wrongdoers to account for their wrongs.^58^ The *telos* of the criminal law demands no less. And no more.

One implication of our argument here is that this view should be rejected. Just as we are more successful morally when we prevent primary rather than (the corresponding) secondary wrongs, so our criminal law is more successful *qua* criminal law when it does the former rather than the latter. The *telos* of the criminal law is thereby more fully realised. In this respect, criminal law and morality have more in common than is often supposed. In another respect, however, they have less. On the popular picture we have opposed, criminal laws that lack counterparts with the same content in morality are, by that very token, defective criminal laws. They are norms that fail to live up to criminal law’s internal morality. Our central argument has been that this is not so. Departures from the mirror principle, far from being condemned by criminal law’s internal morality, are sometimes justified by it. This is so because the instrumental principle is internal to the nature of the criminal law, because that principle sometimes conflicts with the mirror principle, and because those conflicts are sometimes rightly resolved by preferring the former to the latter.

## Footnotes

1 Lon Fuller, *The Morality of Law* (rev edn, Yale University Press 1969).

2 See eg Joseph Raz, *The Authority of Law* (OUP 1979) ch 11; John Finnis, *Natural Law and Natural Rights* (2nd edn, OUP 2011) ch 10; John Gardner, *Law as a Leap of Faith* (OUP 2012) chs 7–9.

3 For relevant citations, see nn 7–8 and 20–2.

4 Fuller (n 1) 33ff.

5 ibid. We borrow the phrase from John Finnis. See Finnis (n 2) 270.

6 Our focus is on substance, not procedure. Whether the mirror principle extends to procedural norms is a question we cannot address here.

7 This thesis is often known as the wrongfulness constraint. Endorsements include Michael Moore, ‘A Tale of Two Theories’ (2009) 28 Criminal Justice Ethics 27, 31–2; Douglas Husak, *Overcriminalization: The Limits of Criminal Law* (OUP 2008) ch 2; AP Simester and Andreas von Hirsch, *Crimes, Harms and Wrongs: On the Principles of Criminalisation* (Hart Publishing 2011) ch 2; RA Duff, *The Realm of Criminal Law* (OUP 2018) 58–70.

8 This second thesis follows from the following conjunction: (i) D should be criminally liable for φing only if D deserves to be censured and/or punished for having φed; and (ii) D deserves to be censured and/or punished for having φed only if D lacks a moral justification or excuse for φing. See eg Michael Moore, *Placing Blame: A Theory of Criminal Law* (OUP 1997) 486; AP Simester, *Fundamentals of Criminal Law: Responsibility, Culpability, and Wrongdoing* (OUP 2021) ch 1.

9 We discuss some examples from English law in the text to nn 45–51.

10 All else being equal. It is perfectly possible, of course, for criminalisation to prevent or ameliorate some wrongs and cause or exacerbate others. In cases like these, whether the instrumental principle is satisfied depends on whether our reasons to do the former defeat our reasons not to do the latter. No doubt this can be a complicated question. But there is no reason to believe that determining which norms of the criminal law are defensible should be thought to be a simple matter.

11 John Gardner, ‘Review of Douglas Husak, *Overcriminalization: The Limits of the Criminal Law*’ (*Notre Dame Philosophical Reviews*, 3 August 2008) <https://ndpr.nd.edu/reviews/overcriminalization-the-limits-of-the-criminal-law/> accessed 21 September 2021.

12 John Gardner, ‘Ethics and Law’ in John Skorupski (ed), *The Routledge Companion to Ethics* (Routledge 2010) 421.

13 Other subscribers to JIDO include Simester and von Hirsch (n 7) 24–9; RA Duff and SE Marshall, ‘“Remote Harms” and the Two Harm Principles’ in AP Simester, Antje du Bois-Pedain and Ulfrid Neumann (eds), *Liberal Criminal Theory: Essays for Andreas von Hirsch* (Hart Publishing 2014) 216, 219–20.

14 Doubts about JIDO have been expressed by others. Here, we try to vindicate those doubts. See eg Andrew Cornford, ‘Rethinking the Wrongness Constraint on Criminalisation’ (2017) 36 Law and Philosophy 615; Re’em Segev, ‘Moral Innocence and the Criminal Law: Non-Mala Actions and Non-Culpable Agents’ (2020) 79 CLJ 549.

15 It might be said that our argument here overlooks the possibility of a licensing regime. Legislators might criminalise use of the cheaper process on condition that the user lacks a licence. Andrew Simester argues that, under such a regime, P would have two options: either (i) get the licence or (ii) claim to be different. Since P’s claim would need to be tested, that claim (and others like it) would be costly for others. Those costs explain why P would have a moral duty to opt for (i). Two brief replies. First: licensing regimes are themselves costly to set up and administer. They introduce new avenues for exploitation and abuse. For these reasons, legislators may be justified in preferring the all use option. In cases where this is so, the argument in the text holds. Second, we can grant that, under a licensing regime, P would have a moral duty not to make costly claims. It does not follow that this duty requires P to get a licence. Imagine P could make use of the cheaper process in secret. If so, P could conform to her duty not to make costly claims without getting a licence, and without P’s use of the cheaper process encouraging misuse by Q. For Simester’s discussion, see Simester (n 8) 240–2.

16 This is not to deny that it may be wrong to breach a legal duty for reasons other than those that justify the duty’s existence. It is only to claim that such reasons obtain contingently. For extended discussion, see Cornford (n 14); Segev (n 14).

17 See eg Husak, *Overcriminalization* (n 7) 78; Moore, ‘A Tale of Two Theories’ (n 7) 31–2; Duff, *Realm* (n 7) 57.

18 See eg Victor Tadros, *Wrongs and Crimes* (OUP 2016) 98–101 and ch 17; Cornford (n 14) 634–8.

19 Michael Moore, ‘Responses and Appreciations’ in Kimberly Kessler Ferzan and Stephen J Morse (eds), *Legal, Moral, and Metaphysical Truths: The Philosophy of Michael S Moore* (OUP 2016) 345–7.

20 RA Duff, ‘Perversions and Subversions of Criminal Law’ in RA Duff and others (eds), *The Boundaries of Criminal Law* (OUP 2010) 92.

21 Husak, *Overcriminalization* (n 7) 57. The reference to Fuller is significant because Fuller explicitly accepts that law’s external morality can justify departures from its internal morality.

22 Douglas Husak, ‘Wrongs, Crimes and Criminalization’ (2019) 13 Criminal Law and Philosophy 393, 398; Moore, ‘Responses’ (n 19) 357; RA Duff, ‘Defending the Realm of Criminal Law’ (2020) 14 Criminal Law and Philosophy 465, 483–4.

23 These ends are given different names by different writers. Depending on who you read, they constitute law’s point, its purpose, its function or its aim.

24 Fuller (n 1) 33–94; Raz (n 2) 212–19.

25 Fuller (n 1) 130.

26 Ronald Dworkin, *Law’s Empire* (Harvard UP 1986) 93.

27 Finnis (n 2) ch 10.

28 Dworkin (n 26) 103–4; Finnis (n 2) 276.

29 HLA Hart, *The Concept of Law* (3rd edn, OUP 2012) 249.

30 We borrow this point from John Gardner. See Gardner, *Law as a Leap of Faith* (n 2) 208.

31 Versions of the argument from censure are found in Simester and von Hirsch (n 7) 19–20; Duff, *Realm* (n 7) 55–7.

32 Moore, *Placing* (n 8) 28.

33 Duff, ‘Defending’ (n 22) 496–7.

34 Moore, *Placing* (n 8) 28, 91–2, 486; Michael Moore, ‘Legal Moralism Revisited’ (2017) 54 San Diego L Rev 441.

35 See, inter alia, RA Duff, *Answering for Crime* (Hart Publishing 2007) 216–23; Antony Duff and others, *The Trial on Trial*: *Towards a Normative Theory of the Criminal Trial*, vol III (Hart Publishing 2007) 142–8; RA Duff, ‘Responsibility, Citizenship, and Criminal Law’ in RA Duff and Stuart P Green (eds), *Philosophical Foundations of Criminal Law* (OUP 2011) 127–30.

36 Joseph Raz, *Practical Reasons and Norms* (OUP 1999) 159.

37 Hart (n 29) 197.

38 ibid 198.

39 It is true, of course, that all else need not be equal. A government might obtain exceptionless conformity by all manner of nefarious means. We can stipulate, however, that this is not so in our imagined society: that its legal (and other governmental) institutions are scrupulous in conforming to the demands of the rule of law.

40 We assume that, where X has a *telos*, success for X consists in X serving that *telos*. It follows that, if X can serve its justifying end(s) only if Y, Y is a pre-condition of X’s success.

41 Moore, *Placing* (n 8) 28–9.

42 To take but one, the reduction of crime is one of five statutory purposes to which sentencing courts must have regard in England and Wales. See s 57 of the Sentencing Act 2020.

43 Moore, *Placing* (n 8) 28–9, 191–3, 246–7, 486; Michael Moore, *Act and Crime: The Philosophy of Action and its Implications for Criminal Law* (OUP 2010) 178–83, 243–4.

44
*Gammon* [1985] 1 AC 1, 14.

45 See eg *Ashley* [2008] UKHL 25, [2008] 1 AC 962 [17] and [51].

46 We borrow the example from Paul Robinson, *Criminal Law Defenses* (West 1984) 78.

47 Marcia Baron, ‘Gender Issues in Criminal Law’ in John Deigh and David Dolinko (eds), *The Oxford Handbook of Philosophy of Criminal Law* (OUP 2011) 363.

48
*Southwark LBC v Williams* [1971] 2 WLR 467 (CA) 473.

49
*Dudley and Stephens* [1884] 14 QBD 273 (QB) 287.

50 ibid 288. The fragment is from *Paradise Lost*. Satan—Milton’s fiend—concludes his plea in the following terms: ‘Thank him who puts me loath to this revenge/On you who wrong me not for him who wrongd./And should I at your harmless innocence/Melt, as I doe, yet public reason just,/Honour and Empire with revenge enlarg’d,/By conquering this new World, compels me now/To do what else though damnd I should abhorre.’

51
*Dudley* (n 49) 288. Similar points could be made about other defences. To mention just one, consider the tightening of the requirements of duress brought about by the House of Lords in *Hasan* [2005] UKHL 22, [2005] 2 AC 467. The rationale given by the majority? ‘The policy of the law must be to discourage association with known criminals’ ([38]).

52 See text to n 31.

53 None of this is to say that the argument from censure is itself unsound. As Feinberg recognised, one can consistently endorse that argument while also endorsing the argument we made in the previous section. One can hold both that (i) criminal law’s modality—the censorious nature of its means—implies that those convicted of crimes ought to be morally blameworthy and that (ii) criminal law’s *telos* is (at least partly) preventive: Joel Feinberg, *The Moral Limits of the Criminal Law, vol 4: Harmless Wrongdoing* (OUP 1990) 150.

54 Moore, *Placing* (n 8) 91, 154, 171–2.

55 Duff, ‘Responsibility’ (n 35) 140; RA Duff, ‘Relational Reasons and the Criminal Law’ in Leslie Green and Brian Leiter (eds), *Oxford Studies in Philosophy of Law*, vol 2 (OUP 2013) 205.

56 An objection raised by Duff in *Realm* (n 7) 244.

57 Duff could respond that our reasons to call public wrongdoers to public account are mere first-order reasons. Moore could make a similar claim about our reasons to give wrongdoers their just deserts. Adding norms to the criminal law as a means of conforming to those reasons would not then prevent a wrong. We doubt, however, that these responses have much to recommend them. They imply that the *telos* of the criminal law—its justifying end—is one that political communities have no duty to pursue. Given the valuable resources consumed by systems of criminal law, and the damage they inevitably do to people’s lives, it is hard to believe that the justifying end of such systems could be supererogatory.

58 This is true, at least, for Moore and Duff, both of whom claim that the moral duties discharged by criminal trials and punishments are secondary duties. See Moore, *Placing* (n 8) 170–2; Duff, ‘Responsibility’ (n 35) 140.

